# Stable sub-micrometre high-flux probe for soft X-ray ARPES using a monolithic Wolter mirror

**DOI:** 10.1107/S1600577520007274

**Published:** 2020-07-10

**Authors:** Yasunori Senba, Hikaru Kishimoto, Yoko Takeo, Hirokatsu Yumoto, Takahisa Koyama, Hidekazu Mimura, Haruhiko Ohashi

**Affiliations:** a Light Source Division, Japan Synchrotron Radiation Research Institute, 1-1-1 Kouto, Sayo, Hyogo 679-5198, Japan; b Department of Precision Engineering, School of Engineering, The University of Tokyo, 7-3-1 Hongo, Bunkyo-ku, Tokyo 113-8656, Japan

**Keywords:** focusing optics, Wolter mirror, monolithic, soft X-rays, ARPES

## Abstract

A focusing optics that can provide a sub-micrometre high-flux probe for soft X-ray micrometre-scale angle-resolved photoemission spectroscopy (ARPES) is proposed. A monolithic Wolter-type mirror with a large acceptance, achromatism and small comatic aberration was designed and evaluated. A focused beam size of 0.4 µm (vertical) × 4 µm (horizontal), a high throughput of 59% and a high tolerance of 1.6 mrad to the pitching error were realized at a photon energy of 1000 eV. A Wolter-type mirror can be practically employed as a stable sub-micrometre focusing mirror with high throughput in ARPES applications.

## Abstract 2

A monolithic Wolter-type mirror with large acceptance, achromatism and small comatic aberration was designed and evaluated at the soft X-ray beamline BL25SU of SPring-8.

## 1. Introduction   

Soft X-ray angle-resolved photoemission spectroscopy (ARPES) is a powerful technique used to evaluate the electronic structures of solid surfaces in the momentum space. The photoexcitation cross section of the valence electron is small in the soft X-ray region; furthermore, the electron energy and emission angle need to be accurately resolved in ARPES. Hence, soft X-ray ARPES requires a monochromatic beam with a high resolving power and flux in a wide range of energies. The spectra obtained by ARPES experiments provide spatial-averaged information of sample surfaces. A large probe beam can degrade the resolutions of the electron energy and angular distribution if the sample surface is not uniform and/or is rough. A small probe beam is essential for high-quality spectra, because exotic materials generally have small homogeneous surface areas, and poorly cleaved samples have small flat surface areas, typically on the order of micrometres. Therefore, ARPES apparatus is equipped with focusing optics to realize a small and highly stable probe beam.

Many soft X-ray ARPES beamlines equipped with various focusing optics are in operation in synchrotron radiation facilities. Diffractive optics, namely Fresnel zone plates (FZP), have been used to realize a beam size on the order of sub-micrometres (ALSNews, 2016 ▸; Avila *et al.*, 2017 ▸). The FZP can realize a focusing size of less than 100 nm with very short working distances (on the order of a few tens of millimetres or shorter) and it can be easily aligned. However, it is a chromatic optics with low acceptance and efficiency. By contrast, a reflective mirror is an example of achromatic optics and has a larger acceptance and efficiency than the FZP. Its working distance, typically on the order of a few hundred millimetres or more, is longer than that of the FZP. The Kirkpatrick–Baez (KB) mirror (ALSNews, 2016 ▸; Reininger *et al.*, 2012 ▸; Siewert *et al.*, 2019 ▸) and toroidal mirror (Strocov *et al.*, 2014 ▸) with a focusing size on the micrometre order are commonly used focusing optics. Single-reflection capillary mirror optics have been developed to achieve a sub-micrometre focusing size (Koch *et al.*, 2018 ▸). However, the acceptance is smaller than that of other reflective mirrors. Their focusing performance deteriorates with increasing source size because of comatic aberration.

The Wolter-type focusing mirror (Wolter, 1952 ▸) is an example of achromatic optics, roughly satisfying Abbe’s sine condition. Hence, this optics has a small comatic aberration and can be used as an imaging optics. However, it is difficult to fabricate a Wolter-type mirror, because it comprises two 2D aspherical surfaces with a small curvature in the sagittal direction. Advanced KB (AKB) optics is a type of Wolter-type optics (Matsuyama *et al.*, 2017 ▸), comprising two pairs of 1D Wolter optics realized by elliptical and hyperbolic cylindrical mirrors. Because AKB optics comprises four mirrors, the total reflectivity is low, and the alignment of the mirrors is complex. A tubular rotationally symmetric Wolter mirror is used for the imaging optics in the fields of astronomy (Raimondi & Spiga, 2015 ▸) and synchrotron radiation (Takeuchi *et al.*, 2001 ▸). A high-precision tubular mirror has recently been developed (Egawa *et al.*, 2019 ▸); however, the substrate material (Ni) is magnetic, which is unsuitable for ARPES.

This paper proposes a monolithic Wolter-type focusing mirror to provide a bright sub-micrometre probe applicable in a wide range of energies for micro-ARPES. A Wolter mirror with large acceptance, achromatism, long working distance, high demagnification and small comatic aberration is adequate for ARPES experiments. The adjusting mechanism and mirror alignment are improved by fabricating the ellipsoid and hyperboloid surfaces on a single substrate. However, the figure error of a steep aspherical mirror should be ideally less than a few nanometres, from the Rayleigh criterion (Born & Wolf, 1999 ▸), for 1 keV sub-micrometre focusing. This figure error corresponds to a slope error of several hundred nano­radians or less. Furthermore, small deviations in the angle and position between the ellipsoid and hyperboloid surfaces are required. The Wolter mirror substrate was fabricated by Natsume Optical Corporation.

The performance test results evaluated using soft X-rays of 300 eV and 1000 eV were sent to the manufacturer. They fabricated the mirror using conventional polishing technology and measuring techniques generally used for small aspheric lenses with high precision.

This paper reports the design of the monolithic Wolter mirror and its performance, evaluated in terms of focused beam size, transmission efficiency and tolerance to alignment error.

## 2. Optical design   

**Figure 1:**
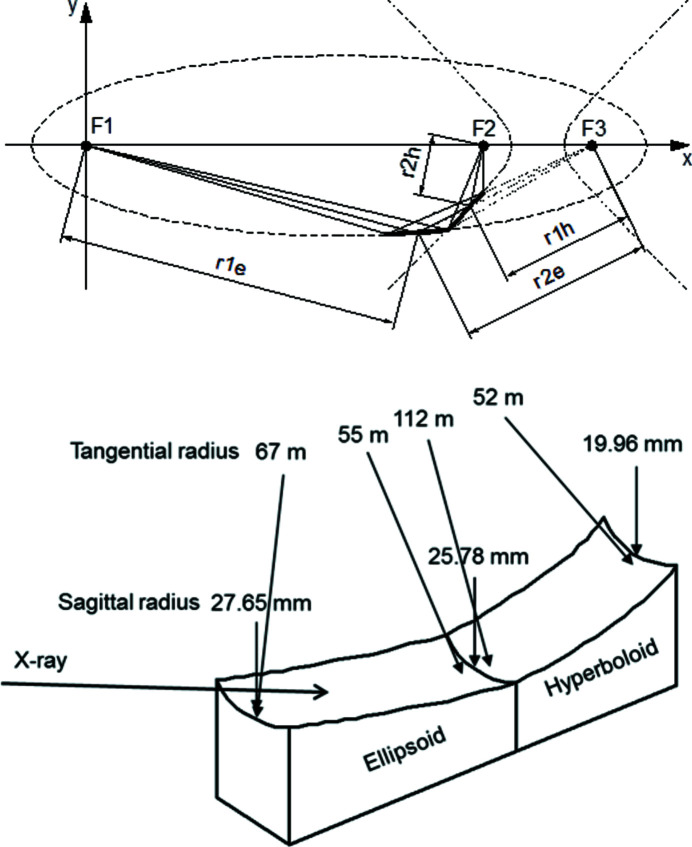
Optical layout (top) and schematic view (bottom) of the monolithic Wolter type-I mirror. The radii of curvature in the sagittal and tangential directions are shown in the schematic view.

A large-acceptance and high-demagnification focusing optics was designed for a micro-ARPES study at the soft X-ray beamline BL25SU of SPring-8 (Senba *et al.*, 2016 ▸). Fig. 1 ▸ shows the optical layout and schematic view of the monolithic Wolter type-I mirror designed here, which consists of ellipsoid and hyperboloid surfaces on a substrate. One of the focal points of ellipsoid F1 is located at the source point. The light reflected by the ellipsoid is focused at the other focal point F3. The focal point of ellipsoid F3 coincides with that of the hyperboloid. The reflected light on the ellipsoid is finally focused at the other focal point F2 after reflecting at the hyperboloid.

Table 1 ▸ lists the parameters of the mirror. The glancing angles at the centers of the two surfaces are 1.2° and 1°, which are determined in such a way that the total reflectivity is higher than 55% at a photon energy of 1000 eV. The working distance is 250 mm. The mirror is placed to reflect in the horizontal plane, thus attaining a large vertical acceptance (V) of 15 mm and a horizontal acceptance (H) of 2 mm. The mirror has a magnification of 1/38. The allowances of the relative angles for pitching, rolling and yawing between the ellipsoid and hyperboloid surfaces are estimated to be ±50 µrad, ±500 µrad and ±10 µrad, respectively, from the results of the ray-tracing calculations obtained using *SHADOW* (Sanchez del Rio *et al.*, 2011 ▸). To satisfy the Rayleigh criterion at 1000 eV, the figure error is determined to be 3 nm r.m.s.

The light source of the focusing optics is typically located on the exit slit of a grating monochromator. The typical source size at BL25SU branch-a in the vertical and horizontal directions is on the order of a few tens of micrometres to several hundred micrometres; hence, a focusing size of sub-µm × 10 µm can be expected. The incident beam size at the focusing optics position is on the order of a few millimetres in the vertical direction and approximately 1 mm in the horizontal direction, which is small enough to be allowed through the mirror opening with dimensions of 15 mm (V) × 2 mm (H).

## 3. Experimental setup   

**Figure 2:**
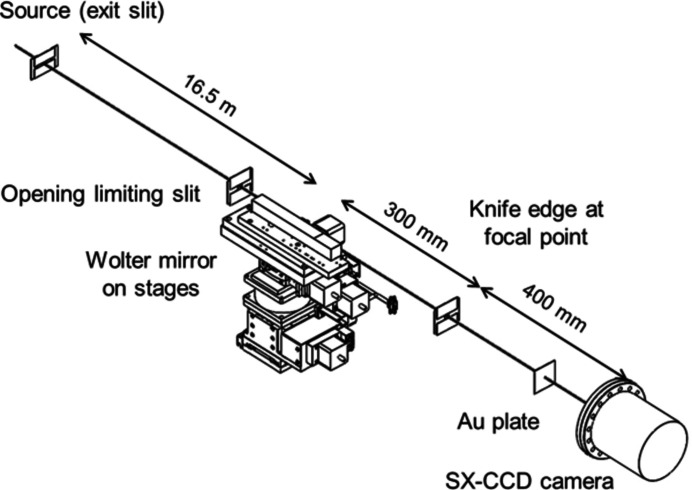
Schematic view of the performance evaluation apparatus.

Fig. 2 ▸ shows the performance evaluation apparatus installed 16.5 m from the exit slit of BL25SU branch-a. The distance between the exit slit and the mirror was 3.1 m longer than the designed entrance arm length of the mirror. Therefore, the magnification of this setup is 1/55. The mirror was mounted on three-axis angular and two-axis positioning stages. The knife edges installed at the focusing position are mounted on a piezo-driven linear stage, which can move in the vertical, horizontal and longitudinal directions. The knife edge is prepared from an Au-coated chemical-etched Si wafer. The beam intensity is measured as the drain current of the Au plate installed following the focusing position. A reflected beam image downstream of the focusing position is captured using a back-illuminated CCD camera (PIXIS-XO-2048B, Princeton Instruments). The setup of the varied-line-spacing plane-grating monochromator is as follows. A spherical mirror M21a and grating with center groove densities of 300 lines mm^−1^ and 600 lines mm^−1^ were used for 300 eV and 1000 eV, respectively. The exit slit width of the monochromator was set to 20 µm in the vertical direction, which corresponds to an energy resolving power (*E*/Δ*E*) of 10 000.

## 4. Results and discussion   

**Figure 3:**
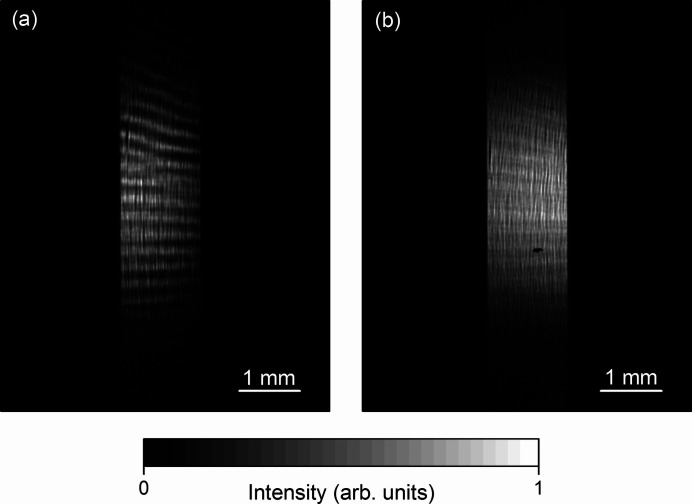
Reflected beam image measured for (*a*) the first received mirror and (*b*) the figure-corrected mirror.

The position and angle adjustment of the mirror and positioning of the knife edge at the focusing position were carried out using the mirror stage, knife-edge scanner and CCD camera. Fig. 3 ▸ shows the reflected beam image measured at 1000 eV. The image of the first received mirror shows clear stripe patterns with a period of 0.3 mm. The fluctuation in the beam intensity was caused by the periodic figure error on the mirror surface in the sagittal direction. The period of the figure error is estimated to be 0.2 mm. The slope errors in the tangential and sagittal directions were improved to 0.4 µrad and 2.7 µrad r.m.s., respectively, by further figure corrections of the mirror. After the corrections, the periodic structures on the image were decreased.

**Figure 4:**
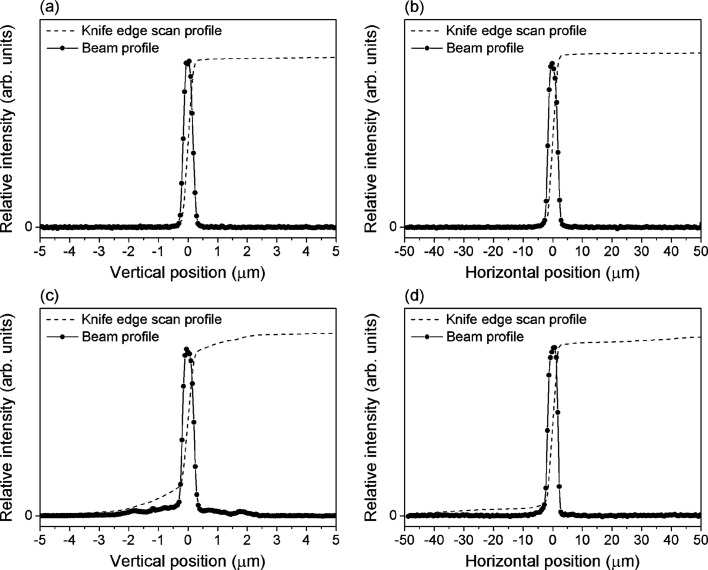
Intensity profile (broken line) and differentiation (circles) measured using a knife-edge scanner in (*a*) the vertical direction and (*b*) the horizontal direction at 300 eV, and in (*c*) the vertical direction and (*d*) the horizontal direction at 1000 eV.

The focused beam profiles were measured for the coated Wolter mirror. The mirror substrate was coated with Au on Cr (a bonding material) by DC magnetron sputtering. The thicknesses of the Au and Cr layers were 30 nm and 5 nm, respectively. Fig. 4 ▸ shows the focused beam profiles measured at 300 eV and 1000 eV. Each profile is obtained from the differentiation of the Au drain current with respect to the knife-edge position.

The source sizes in the vertical and horizontal directions are 20 µm and 200 µm, respectively. The focused beam profiles measured for 300 eV were fitted using a single Gaussian profile, the full width at half-maximum (FWHM) of which is 0.32 µm in the vertical direction and 3.4 µm in the horizontal direction. The areas in the FWHM in the vertical profile (77%) and in the horizontal profile (74%) are largely the same as the area of the ideal Gaussian distribution (76%). This shows that the focused beam has a near-ideal Gaussian profile. The estimated beam sizes are approximately equal to the calculated values using source size and magnification.

Small peaks around the main peak are observed in the vertical profile measured for 1000 eV. The beam sizes are slightly wider than those measured for 300 eV; the sizes in the vertical and horizontal directions are 0.37 µm and 3.6 µm, respectively. The areas in the FWHM in the vertical profile (61%) and in the horizontal profile (70%) are slightly smaller than that of the ideal Gaussian distribution. This can be attributed to the small figure error on the mirror surface.

The throughput is evaluated as the ratio of the reflected beam intensity to the direct beam intensity. The estimated throughputs of 62% for 300 eV and 59% for 1000 eV are in good agreement with the calculated reflectivity. Therefore, the acceptance of the mirror is considered to be almost 100%.

**Figure 5:**
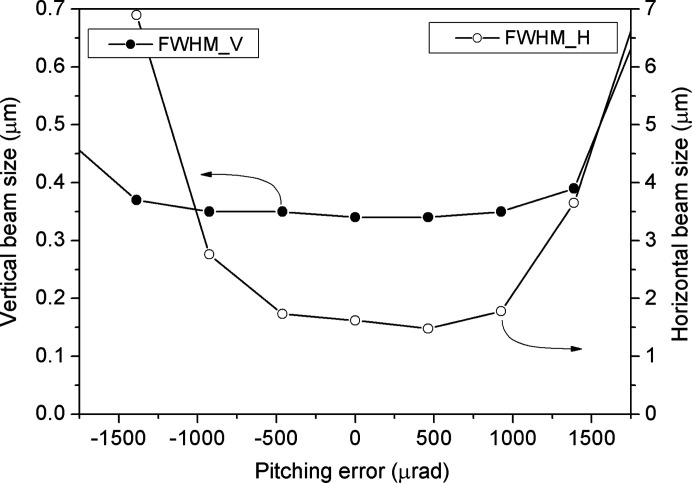
Relationship between the relative pitching error and focused size measured at 1000 eV with a source size of 20 µm × 100 µm.

Fig. 5 ▸ shows the pitching error dependence of the focused beam size measured at 1000 eV. The source sizes in the vertical and horizontal directions are 20 µm and 100 µm, respectively. The pitching angle is varied in the range ±1.5 mrad while the distance between the mirror and the knife edge is fixed at the best focusing distance. The beam size remains largely unchanged in the pitching error range ±800 µrad. This wide tolerance indicates that the Wolter-type optics has a small comatic aberration. Therefore, the focusing size is highly stable, and the optics can be easily aligned.

This Wolter mirror has been installed in the ARPES apparatus at BL25SU of SPring-8 (Kinoshita *et al.*, 2019 ▸). The focusing sizes at the sample position were roughly evaluated to be 0.4 µm in the vertical direction and 4 µm in the horizontal direction. Here, the source sizes were 20 µm in the vertical and 200 µm in the horizontal, and the photon energy was 750 eV, which are typically used for ARPES application at BL25SU. The probe beam on the sample surface can be almost round with a diameter of ∼5 µm even at a glancing angle of 5°. The photoemission intensity is enhanced by the configuration of grazing incidence (Strocov, 2013 ▸). Using this small probe, ARPES measurements can be performed on small cleaved surfaces. The results of some demonstrative ARPES measurements on a cleaved surface as small as a few tens of micrometres will be reported elsewhere (Muro *et al.*, in preparation).

## 5. Conclusions   

A monolithic Wolter-type mirror employed as the soft X-ray focusing optics was designed and evaluated. The mirror exhibits large acceptance with dimensions of 15 mm (V) × 2 mm (H), high demagnification of 1/55 and a long working distance of 250 mm. The focused beam has vertical and horizontal dimensions of 0.32 µm and 3.4 µm, respectively, and a reflectivity of 62% at 300 eV. A high tolerance to the pitching error that can be attributed to small comatic aberration has been confirmed. The monolithic Wolter mirror has been used in the micro-ARPES apparatus at BL25SU of SPring-8. This monolithic Wolter mirror can be applied to hard X-ray regions by optimizing the optical layout with a small incident angle on the order of a few microradians. The mirror has the potential to achieve sub-micrometre focusing with high stability, high throughput and easy alignment.

The small satellite structures around the main focusing profile at 1000 eV do not significantly affect the focusing size. This is caused by the figure error on the mirror surface and should be studied in greater detail in future work.

## Figures and Tables

**Table 1:** Parameters of the monolithic Wolter type-I mirror

Shape			Monolithic Wolter
Material			SiO_2_
Deflection of X-ray			Horizontal
Outer dimensions (mm)			210 × 25 × 25 (L × W × T)
Useful area (mm)			200 × 15 (L × W)
Length of ellipsoid (mm)			98
Length of hyperboloid (mm)			102
Focal parameters			
Ellipsoidal part			
Incident angle (°)			1.2
r1e (mm)			13400
r2e (mm)			670.417
Major axis (mm)			7035.209
Minor axis (mm)			62.76995
Hyperboloidal part			
Incident angle (°)			1.0
r1h (mm)			562.129
r2h (mm)			300.000
Major axis (mm)			131.065
Minor axis (mm)			7.16694
